# Supplementary material for: Structure and function of H+/K+ pump mutants reveal Na+/K+ pump mechanisms
Source: Nat Commun. 2022 Sep 9;13:5270. doi: 10.1038/s41467-022-32793-0 (PMC9463140; doi:10.1038/s41467-022-32793-0)
Supplement: Supplementary file 1 — Supplementary Information [file 41467_2022_32793_MOESM1_ESM.pdf]

Supplementary Information for

Structure and function of H^+/K^+ pump mutants reveal Na^+/K^+ pump mechanisms

Victoria C. Young[1†], Hanayo Nakanishi[2†], Dylan J. Meyer[1], Tomohiro Nishizawa[3], Atsunori Oshima[2,4,5], Pablo Artigas[1*], Kazuhiro Abe[2,4*]

[1]Department of Cell Physiology and Molecular Biophysics, Center for Membrane Protein Research, Texas Tech University Health Sciences Center, Lubbock, TX, USA.

[2]Cellular and Structural Physiology Institute, Nagoya University, Nagoya 464-8601, Japan

[3]Graduate School of Medical Life Science, Yokohama City University, Tsurumi, Yokohama, 230-0045, Japan

[4]Graduate School of Pharmaceutical Sciences, Nagoya University, Nagoya 464-8601, Japan

[5]Institute for Glyco-core Research (iGCORE), Nagoya University, Nagoya 464-8601, Japan

*Corresponding author. Email: pablo.artigas@ttuhsc.edu or kabe@cespi.nagoya-u.ac.jp

†Authors contributed equally to this work.

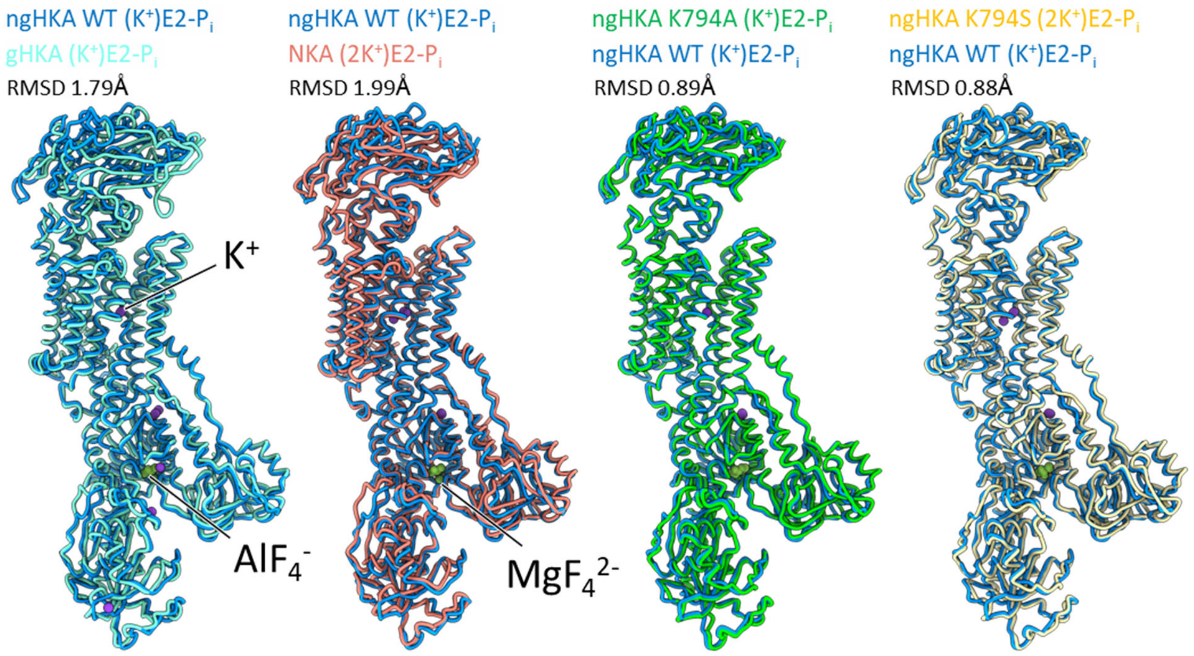

Supplementary Figure 1| Comparisons of the molecular conformations

The overall molecular conformations of $(K^+)E2\text{-}P_i$ state of ngHKA WT (blue), gHKA (cyan, 6jxh), NKA (brown, 2zxe), ngHKA K794A (green) and ngHKA K794S (yellow) are indistinguishable. The relatively high RMSD values when comparing ngHKA with gHKA (1.79 Å) and NKA (1.99 Å) may be due to their different α subunit and/or to the different β-subunit in the complexes (NKA β1 (ATP1B1) for ngHKA and NKA, or gastric β (ATP4B) for gHKA). Bound ligands are indicated as spheres with different colors (K^+: purple, AlF_4 or MgF_4: light green). All models are aligned by their immobile TM7-10 region of the α subunit.

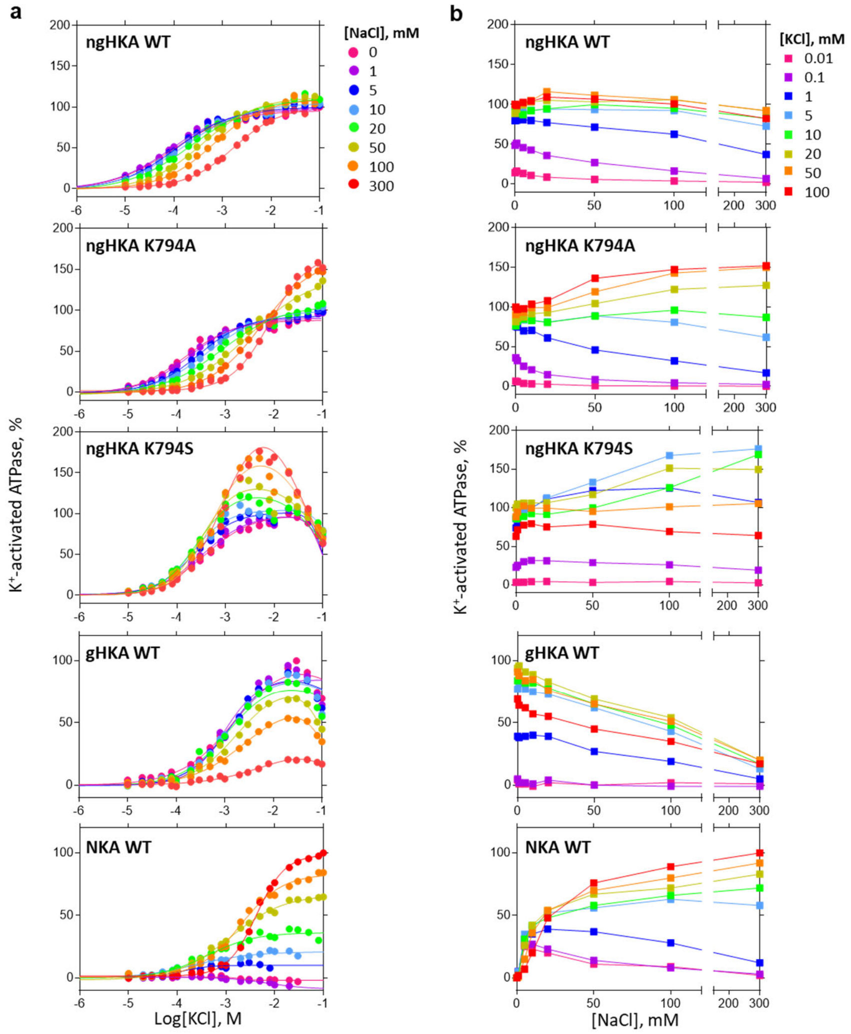

Supplementary Figure 2| ATPase activities of ngHKA Lys794 mutants

(**a**) K^+-dependent ATPase activity of the membrane fractions in the presence of different [Na^+] (color coded in the figure). The specific activity was normalized as 100% of the maximum activity in the absence of Na^+ in the case of WT-, K794A-, K794S-ngHKA and gHKA (the maximum K^+-sensitive specific activities are as follows; 4.1 μmol/mg/h for WT-ngHKA at 80 mM KCl, 15.6 μmol/mg/h for K794A-ngHKA at 100 mM KCl, 7.2 μmol/mg/h for K794S-ngHKA at 20 mM KCl, 2.7 μmol/mg/h for gHKA at 30 mM KCl). For NKA, the maximum activity in the presence of Na^+ was normalized as 100% (the maximum K^+-sensitive specific activity is, 7.8 μmol/mg/h at 100 mM KCl and 300 mM NaCl). Data were fit with single- or double-component Hill's equation (Eq. 3, lines). Activity in the absence of K^+ and Na^+ were set as blanks. (**b**) Data obtained at the indicated [K^+] plotted as a function of [Na^+].

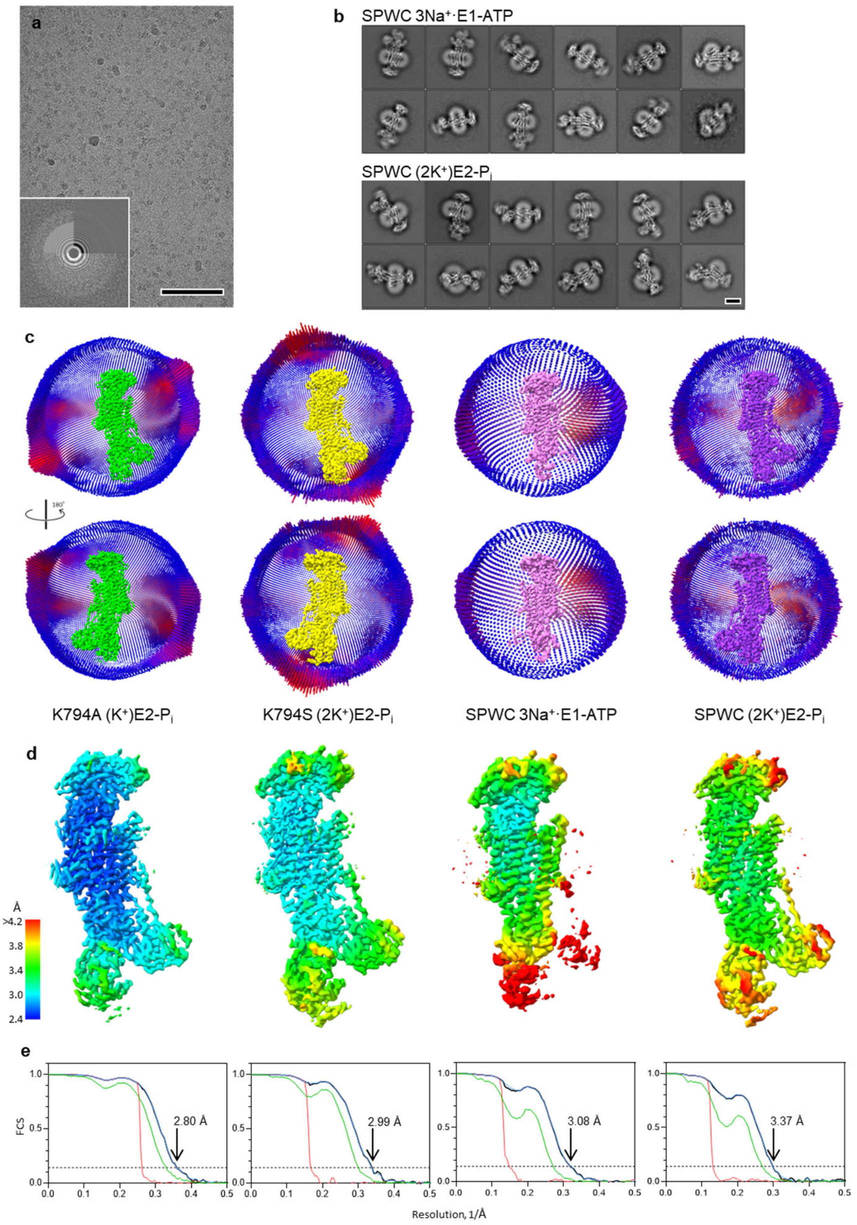

Supplementary Figure 3| Cryo-EM analysis of ngHKA mutants

(**a**) A representative cryo-EM image of SPWC mutant in NaE1AMPPCP state (3,627 micrographs) and its Fourier transform. Bar, 50 nm. (**b**) Representative 2D-class averages obtained from SPWC-ngHKA in the $3Na^{+}\cdot E1$-ATP and in the $(2K^{+})E2\text{-}P_i$ states. Bar, 5 nm. (**c**) Angular distribution plot of particles included in the final 3D reconstruction. The number of views at each angular orientation is represented by the length and color of cylinders. Red indicates more views. (**d**) Final reconstruction map colored by local resolution as calculated by Relion3.1. (**e**) FSC plot used for resolution estimations (black: corrected, blue: masked, green: unmasked, red: phase randomized).

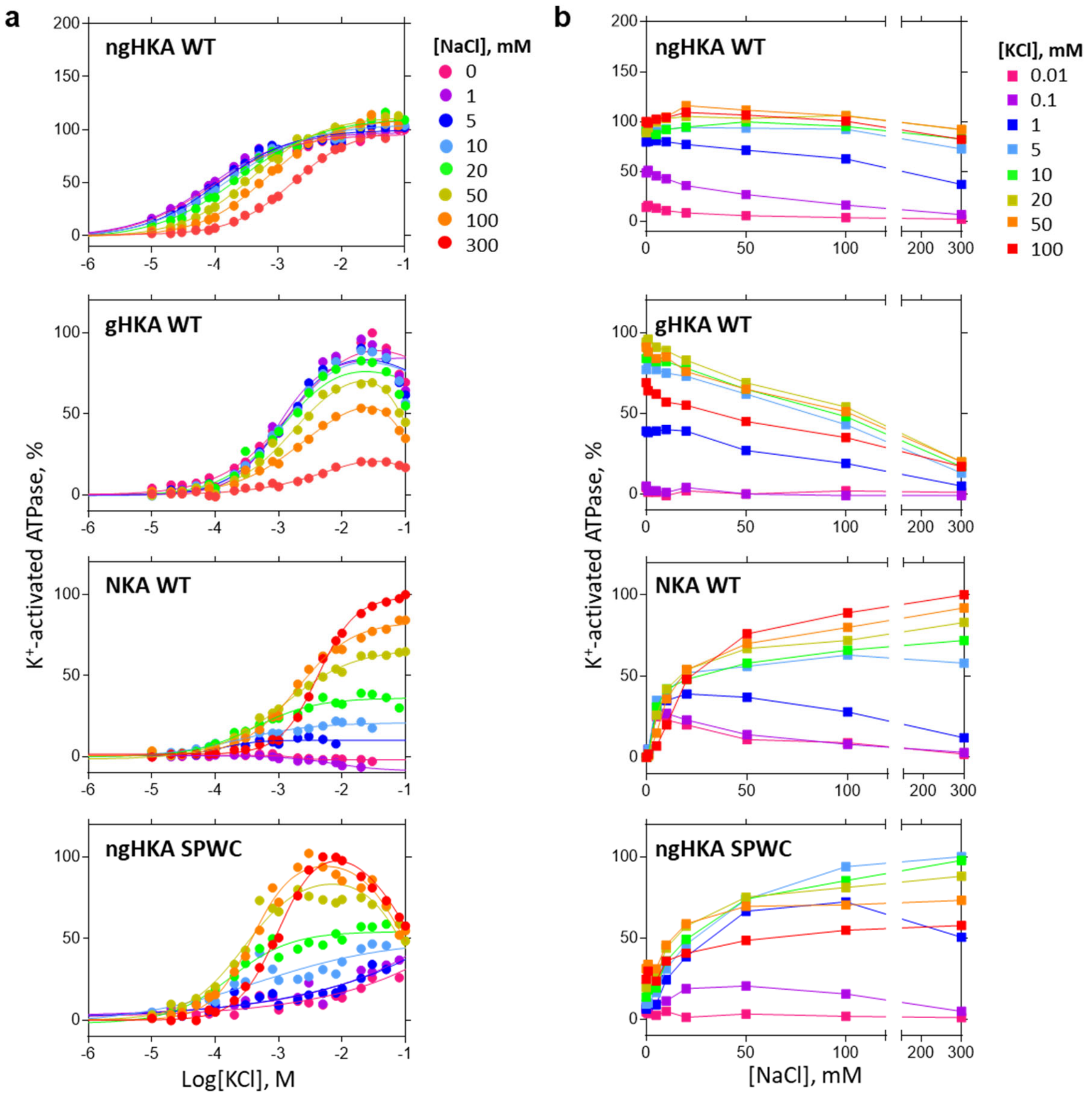

Supplementary Figure 4| ATPase activities of various ATPases studied

(**a**) K^+-dependent ATPase activity of the membrane fractions in the presence of different [Na^+] (color coded in the figure). The specific activity was normalized as 100% of the maximum activity in the absence of Na^+ in the case of WT ngHKA and gHKA. For SPWC-ngHKA and NKA, the maximum activity in the presence of Na^+ was normalized as 100% (the maximum K^+-sensitive specific activities are 1.0 μmol/mg/h for SPWC-ngHKA at 5 mM KCl and 300 mM NaCl). Data were fit with single- or double-component Hill's equation (Eq. 3, lines). Activity in the absence of K^+ and Na^+ were set as blanks (see Methods). (**b**) Data obtained at the indicated [K^+] plotted as a function of [Na^+].

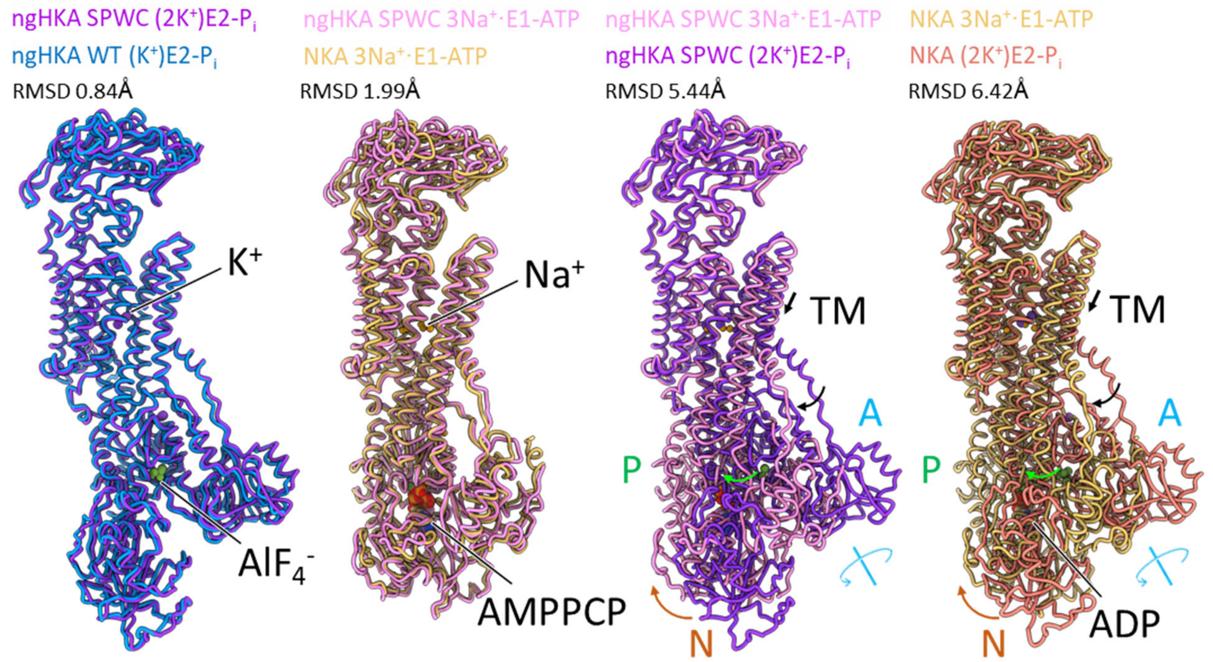

Supplementary Figure 5| Comparisons between SPWC ngHKA and NKA

The overall conformations of $(K^+)E2\text{-}P_i$ state of ngHKA WT (blue) and SPWC-ngHKA (purple) are indistinguishable. The overall conformations of SPWC-ngHKA in $3Na^+\cdot E1$-ATP state (pink) and NKA $(3Na^+)$E1P-ADP (wheat, 3wgu) are also practically indistinguishable. For comparison, the overall conformations E2-P_i and E1-nucleotide are shown for both SPWC-ngHKA and NKA. Displacement of cytoplasmic domains and TM helices are indicated by arrows (from $(2K^+)E2\text{-}P_i$ to $3Na^+\cdot E1$-ATP). Bound ligands are spheres with different colors (Na^+: orange, K^+: purple, AlF_4 or MgF_4: light green, ADP or AMPPCP: CPK coloring).

	**ngHKA WT (K^+)E2-P_i state** [illegible]
**Data collection**	
Resolution (Å)	48.06 – 3.3 (3.42-3.3) [1]
Space group	$P\,2_1\,2_1\,2_1$
Cell dimensions	
a, b, c (Å)	75.97, 109.06, 267.67
α, β, γ (˚)	90, 90, 90
R_{merge}	0.049 (0.83)
R_{pim}	0.049 (0.83)
I/σI	8.36 (0.68)
C/C1/2	1 (0.786)
Completeness (%)	72.54 (14.29)[2]
Redundancy	2.0 (2.0)
**Refinement**[3]	
Resolution (Å)	48.06 – 3.3 (3.42-3.3)
No. of reflections	68,181 (6,682)
R_{work}/ R_{free} (%)	25.8/31.3 (46.6/50.3)
Wilson B-factor	49.43
No. of atoms	
Protein	10,019
Ligands	21
Average B-factor	44.49
Protein (Å^2)	44.44
Ligands (Å^2)	66.66
R.m.s deviations	
Bond lengths (Å)	0.011
Bond angles (˚)	1.39

Supplementary Table 1| Data collection and refinement statistics of X-ray crystallography

[1]Statistics for the highest-resolution shell are shown in parentheses.

Construct	state	site	Amino acid	atom	distance	valence
WT	(K$^+$)E2-Pi	II (K$^+$)	Val341	O	2.81	0.15
			Ala342	O	2.84	0.13
			Val344	O	2.72	0.20
			Glu346	Oε1	2.74	0.18
			Asn795	Oδ1	3.17	0.05
			Glu798	Oε1	3.04	0.07
			Asp823	Oδ1	2.7	0.21
				Total valence		0.99
K794A	(K$^+$)E2-Pi	II (K$^+$)	Val341	O	3.01	0.08
			Ala342	O	2.81	0.15
			Val344	O	2.73	0.19
			Glu346	Oε1	3.36	0.03
			Asn795	Oδ1	2.92	0.10
			Glu798	Oε1	3.29	0.03
			Asp823	Oδ1	2.7	0.21
			Asp823	Oδ1	3.19	0.05
			H2O	O	3.59	0.02
				Total valence		0.86
K794S	(2K$^+$)E2-Pi	I (K$^+$)	Thr791	O	2.82	0.14
			Ser794	Oγ1	2.67	0.23
			Asn795	Oδ1	2.64	0.26
			Asp823	Oδ1	2.76	0.17
			H2O		2.68	0.23
				Total valence		0.81
		II (K$^+$)	Val341	O	2.69	0.22
			Ala342	O	2.65	0.25
			Val344	O	2.92	0.10
			Glu346	Oε1	3.92	0.01
			Asn795	Oδ1	2.87	0.12
			Glu798	Oε1	3.2	0.05
			Asp823	Oδ1	2.58	0.32
				Total valence		1.07

Construct	state	site	Amino acid	atom	distance	valence
SPWC	3Na$^+$·E1-ATP	I (Na$^+$)	Ala342	O	2.54	0.15
			Thr791	O	4.19	0.02
			Ser794	Oγ1	3.33	0.05
			Asn795	Oε1	2.33	0.21
			Asp827	Oδ1	3.85	0.02
			H2O	O	2.59	0.13
			H2O	O	3.29	0.05
			H2O	O	4.09	0.02
				Total valence		0.65
		II (Na$^+$)	Val341	O	3.16	0.06
			Ala342	O	3.02	0.07
			Val344	O	2.57	0.14
			Glu346	Oε1	3.65	0.03
				Oε2	4.50	0.01
			Asp823	Oδ1	2.63	0.13
				Oε2	3.02	0.07
			H2O	O	2.59	0.13
				Total valence		0.64
		III (Na$^+$)	Tyr790	O	2.83	0.09
				π	4.00	0.02
			Thr791	O	4.23	0.02
			Thr793	Oγ1	2.25	0.25
			Ser794	Oγ1	3.74	0.03
			Asp827	Oδ1	3.25	0.05
			Gln942	Oε1	2.46	0.17
			Asp945	Oδ1	3.46	0.04
				Total valence		0.66
	(2K$^+$)E2-Pi	I (K$^+$)	Thr791	O	3.01	0.08
			Ser794	Oγ1	2.79	0.16
			Asn795	Oγ1	2.81	0.15
			Asp823	Oγ1	2.82	0.14
			Glu798	Oδ1	4.28	0.00
				Total valence		0.53
		II (K$^+$)	Val341	O	3.68	0.01
			Ala342	O	2.73	0.19
			Val344	O	2.91	0.11
			Glu346	Oε1	4.18	0.00
			Asn795	Oδ1	2.82	0.14
			Glu798	Oε1	2.87	0.12
			Asp823	Oδ1	2.73	0.19
			Asp823	Oδ2	2.87	0.12
				Total valence		0.89

Supplementary Table 2| Coordination geometry and partial valence in the ngHKA

Partial valence calculated for the K^+ and Na^+ ions assigned to each structure. Only oxygen atoms that likely contribute to the cation coordination (approximately within 4 Å) were included for the valence calculation. See Methods for details.

	ngHKA K794A (K$^+$)E2-P_i state	ngHKA K794S (2K$^+$)E2-P_i state	ngHKA SPWC 3Na$^+$·E1-ATP	ngHKA SPWC (2K$^+$)E2-P_i state
PDB ID	7X21	7X22	7X23	7X24
EMDB	EMD-32954	EMD-32955	EMD-32956	EMD-32957
**Data collection**				
Magnification	105,000			
Voltage (kV)	300			
Electron exposure (e^-/ Å^2)	52			
Defocus range (μm)	0.8 – 1.8			
Pixel size (Å)	0.83			
Symmetry imposed	*C1*			
Movies (no.)	3,106	3,141	3,627	4,150
Initial particle images (no.)	1,101,509	873,901	1,010,045	769,755
Final particle images (no.)	186,711	176,596	68,808	58,951
Map resolution (Å)	2.80	2.99	3.08	3.37
FCS threshold	0.143	0.143	0.143	0.143
**Refinement**				
Initial model used (PDB)	7X20	7X20	7X22	7X22
Model resolution (Å)	2.78	3.1	3.38	3.33
FSC threshold	0.5	0.5	0.5	0.5
Map sharpening *B* factor	-81.88	-78.49	-97.14	-89.46
Model composition				
Non-hydrogen	10,167	10,689	9,970	10,286
Protein residues	1,263	1,263	1,262	1,263
Waters	18	1	7	0
Ligands	1ALF,1MG,1NAG,2K,3PCW,2CLR	1ALF,1MG,1NAG,3K,6PCW,2CLR	1ACP,1MG,3NA	1ALF,1MG,1NAG,3K,5PCW,3CRL
B factor (Å^2)				
Protein	64.71	100.51	17.12	38.01
R.m.s. deviations				
Bond length (Å)	0.03	0.03	0.003	0.003
Bond angles (°)	0.622	0.587	0.539	0.651
Validation				
MolProbity score	1.68	2.13	1.64	1.72
Clashscore	7.35	25.95	9.19	8.29

Supplementary Table 3| Cryo-EM data collection, processing, refinement and validations
