# Supplementary material for: Structure and function of H+/K+ pump mutants reveal Na+/K+ pump mechanisms
Source: Nat Commun. 2022 Sep 9;13:5270. doi: 10.1038/s41467-022-32793-0 (PMC9463140; doi:10.1038/s41467-022-32793-0)
Supplement: Supplementary file 3 — Description of Additional Supplementary Files [file 41467_2022_32793_MOESM3_ESM.pdf]

File name: Supplementary Movie 1

Description: **Conformational change of SPWC-ngHKA.** Whole structure of SPWC-ngHKA (*left, membrane plane view*) and its TM helices (*right,* extracellular side view) as the pump transitions from $(2K^{+})E2\text{-}P_{i}$ (transparent purple) to $3Na^{+}\cdot E1\text{-}ATP$ (pink) states. See also Fig. 8.
