# Supplementary material for: Structure and function of H+/K+ pump mutants reveal Na+/K+ pump mechanisms
Source: Nat Commun. 2022 Sep 9;13:5270. doi: 10.1038/s41467-022-32793-0 (PMC9463140; doi:10.1038/s41467-022-32793-0)
Supplement: Supplementary file 5 — Reporting Summary [file 41467_2022_32793_MOESM5_ESM.pdf]

nature portfolio

Corresponding author(s): Pablo Artigas, Kazuhiro Abe

Last updated by author(s): 2022/7/29

Reporting Summary

Nature Portfolio wishes to improve the reproducibility of the work that we publish. This form provides structure for consistency and transparency in reporting. For further information on Nature Portfolio policies, see our Editorial Policies and the Editorial Policy Checklist.

Statistics

For all statistical analyses, confirm that the following items are present in the figure legend, table legend, main text, or Methods section.

n/a	Confirmed	
☐	☒	The exact sample size (*n*) for each experimental group/condition, given as a discrete number and unit of measurement
☐	☒	A statement on whether measurements were taken from distinct samples or whether the same sample was measured repeatedly
☒	☐	The statistical test(s) used AND whether they are one- or two-sided<br>*Only common tests should be described solely by name; describe more complex techniques in the Methods section.*
☒	☐	A description of all covariates tested
☒	☐	A description of any assumptions or corrections, such as tests of normality and adjustment for multiple comparisons
☐	☒	A full description of the statistical parameters including central tendency (e.g. means) or other basic estimates (e.g. regression coefficient) AND variation (e.g. standard deviation) or associated estimates of uncertainty (e.g. confidence intervals)
☒	☐	For null hypothesis testing, the test statistic (e.g. *F*, *t*, *r*) with confidence intervals, effect sizes, degrees of freedom and *P* value noted<br>*Give P values as exact values whenever suitable.*
☒	☐	For Bayesian analysis, information on the choice of priors and Markov chain Monte Carlo settings
☒	☐	For hierarchical and complex designs, identification of the appropriate level for tests and full reporting of outcomes
☒	☐	Estimates of effect sizes (e.g. Cohen's *d*, Pearson's *r*), indicating how they were calculated

Our web collection on statistics for biologists contains articles on many of the points above.

Software and code

Policy information about availability of computer code

Data collection	Zoo system (X-ray), SerialEM (v3.8, cryoEM), Warner OC-725C amplifier with Molecular Devices Digidata 1440 (electrophysiology)
Data analysis	KAMO, XDS (March 15, 2019), ccp4 (v 7.0.078). PHENIX 1.18, Relion3.2, MotionCor2.1, CTFFIND4, Coot0.9.2, PYMOL 2.3.1, ChimeraX 1.2.2, GraphPad PRISM 4, CLUSTALW, PClamp, Originlab

For manuscripts utilizing custom algorithms or software that are central to the research but not yet described in published literature, software must be made available to editors and reviewers. We strongly encourage code deposition in a community repository (e.g. GitHub). See the Nature Portfolio guidelines for submitting code & software for further information.

Data

Policy information about availability of data

All manuscripts must include a data availability statement. This statement should provide the following information, where applicable:

- Accession codes, unique identifiers, or web links for publicly available datasets
- A description of any restrictions on data availability
- For clinical datasets or third party data, please ensure that the statement adheres to our policy

Atomic coordinates and cryo EM density maps reported in this study have been deposited in the Protein Data Bank and Electron Microscopy Data Bank as follows;
PDB entries
7X20: Crystal structure of non gastric H,K-ATPase alpha2 in (K+)E2-AlF state

7X21: Cryo-EM structure of non gastric H,K-ATPase alpha2 K794A in (K+)E2-AlF state
7X22: Cryo-EM structure of non gastric H,K-ATPase alpha2 K794S in (K+)E2-AlF state
7X23: Cryo-EM structure of non gastric H,K-ATPase alpha2 SPWC mutant in 3Na+E1-AMPPCP state
7X24: Cryo-EM structure of non gastric H,K-ATPase SPWC mutant in (2K+)E2-AlF state
EMDB entries
EMD-32954: Cryo-EM structure of non gastric H,K-ATPase alpha2 K794A in (K+)E2-AlF state
EMD-32955: Cryo-EM structure of non gastric H,K-ATPase alpha2 K794S in (K+)E2-AlF state
EMD-32956: Cryo-EM structure of non gastric H,K-ATPase alpha2 SPWC mutant in 3Na+E1-AMPPCP state
EMD-32957: Cryo-EM structure of non gastric H,K-ATPase SPWC mutant in (2K+)E2-AlF state

Human research participants

Policy information about studies involving human research participants and Sex and Gender in Research.

Reporting on sex and gender	N/A
Population characteristics	N/A
Recruitment	N/A
Ethics oversight	N/A

Note that full information on the approval of the study protocol must also be provided in the manuscript.

Field-specific reporting

Please select the one below that is the best fit for your research. If you are not sure, read the appropriate sections before making your selection.

☒ Life sciences ☐ Behavioural & social sciences ☐ Ecological, evolutionary & environmental sciences

For a reference copy of the document with all sections, see nature.com/documents/nr-reporting-summary-flat.pdf

Life sciences study design

All studies must disclose on these points even when the disclosure is negative.

Sample size	No statistical method was used to determine sample size
Data exclusions	No data was excluded
Replication	Biochemical assays, including protein expression, thermal stability and ATPase activity were examined at least three times independently, and their average or representative ones were shown in the manuscript. Electrophysiological experiments were repeated more than 3 times and the number of independent repetitions is stated.
Randomization	n/a. Animals or human research participants were not involved in this study. Thus sample were not randomized for the experiments.
Blinding	n/a. Animals or human research participants were not involved in this study. No blinding used in this study.

Reporting for specific materials, systems and methods

We require information from authors about some types of materials, experimental systems and methods used in many studies. Here, indicate whether each material, system or method listed is relevant to your study. If you are not sure if a list item applies to your research, read the appropriate section before selecting a response.

Materials & experimental systems

n/a	Involved in the study	
☐	☒	Antibodies
☐	☒	Eukaryotic cell lines
☒	☐	Palaeontology and archaeology
☒	☐	Animals and other organisms
☒	☐	Clinical data
☒	☐	Dual use research of concern

Methods

n/a	Involved in the study	
☒	☐	ChIP-seq
☒	☐	Flow cytometry
☒	☐	MRI-based neuroimaging

Antibodies

Antibodies used	anti-FLAG M2 affinity resin
Validation	https://www.sigmaaldrich.com/JP/ja/product/sigma/a2220

Eukaryotic cell lines

Policy information about cell lines and Sex and Gender in Research

Cell line source(s)	HEK-293S GnT1- (ATCC CRL-3022)
Authentication	No further authentication was performed for cell lines.
Mycoplasma contamination	Not tested.
Commonly misidentified lines (See ICLAC register)	No commonly misidentified cell lines were used.
